# Clinical Features, Risk Factors, and Therapy of Epithelial Keratitis after Cataract Surgery

**DOI:** 10.1155/2021/6636228

**Published:** 2021-05-06

**Authors:** Yani Wang, Dongfang Li, Wenjie Su, Yunhai Dai

**Affiliations:** ^1^Medical College, Qingdao University, Qingdao, China; ^2^Qingdao Eye Hospital of Shandong First Medical University, Qingdao, China; ^3^State Key Laboratory Cultivation Base, Shandong Provincial Key Laboratory of Ophthalmology, Shandong Eye Institute, Shandong First Medical University and Shandong Academy of Medical Sciences, Qingdao, China

## Abstract

**Purpose:**

The study aimed to assess the clinical characteristics, risk factors, and therapy of epithelial keratitis after cataract surgery.

**Methods:**

Medical data of 89 consecutive patients who developed epithelial keratitis after cataract surgery, including 37 patients with diabetes mellitus (37 eyes) and 52 patients without diabetes mellitus (52 eyes), were retrospectively reviewed. The clinical characteristics, risk factors, and therapy in those patients were evaluated.

**Results:**

The preoperative tear film function determined by the tear breakup time, meibomian gland atrophy score, and low tear meniscus height in diabetic patients was poorer than nondiabetic patients (*P* < 0.001). Of diabetic patients, 83.78% (31/37) had been diagnosed with meibomian gland dysfunction before cataract surgery and treated with topical nonsteroidal anti-inflammatory drugs after cataract surgery for 44.69 ± 10.51 days, compared to 42.31% (22/52) of nondiabetic patients receiving the topical nonsteroidal anti-inflammatory treatment for 33.35 ± 5.16 days (both *P* < 0.001). Epithelial lesions progressed within three to four days following cataract surgery in 59.46% (22/37) of diabetic patients, versus 30.77% (16/52) of the nondiabetic patients (*P*=0.025). Patients with combined meibomian gland dysfunction and epithelial defects accounted for 48.65% (18/37) in the diabetic group and 25.00% (13/52) in the nondiabetic group (*P* < 0.001). In vivo confocal microscopy showed absence of subbasal never fibers in eyes with epithelial defects, and central corneal sensation was also significantly depressed in those eyes, but there was no significant difference between the two groups (*P*=0.227). Corneal ulceration and herpes simplex keratitis were found in 2.70% (1/37) and 5.41% (2/37) of diabetic patients, respectively. Amniotic membrane transplantation was required in 32.43% (12/37) of patients in the diabetic group, and the proportion was higher than 1.92% (1/52) in the nondiabetic group (*P* < 0.001). Average healing time of the corneal epithelium in the diabetic group was 40.62 ± 20.0 days, much longer than 21.74 ± 6.94 days in the nondiabetic group (*P*=0.002).

**Conclusion:**

Epithelial keratitis after cataract surgery in diabetic patients has the characteristics of rapid development, severe epithelial damage, and slow repair of the corneal epithelium. Amniotic membrane transplantation is a good choice for persistent epithelial defects associated with such epithelial keratitis. Attention should be paid to the tear film function and use of topical nonsteroidal anti-inflammatory drugs in patients undergoing cataract surgery.

## 1. Introduction

The incidence of cataract increases with age. Cataract extraction combined with intraocular lens implantation is an effective measure to produce stabilized postoperative outcomes; however, it has often posed challenges to achieving more comfortable vision. Epithelial keratitis (EK) may be caused by abnormalities in corneal epithelial cell regeneration, connection, adhesion, and migration without limbal cell decompensation [1]. It manifests corneal punctate epitheliopathy at early stage and progresses to severe complications such as corneal defects, infection, and ulcers. EK can be misdiagnosed as herpes simplex keratitis (HSK), a rare complication after cataract surgery [2], in its early disease course. Severe EK affects postoperative vision and comfort. Risk factors for EK have been reported to include mechanical, toxic injury, and injudicious use of topical nonsteroidal anti-inflammatory drugs (NSAIDs) [3–5]. In addition, systemic disease such as diabetes mellitus (DM) has a negative impact on ocular surface functions and corneal subbasal nerve fibers [6]. Decreased density and abnormal morphology of subbasal nerve fibers increase the risk of epitheliopathy, and diabetic patients are more likely to develop secondary diabetic keratopathy [7, 8]. As a special diagnostic tool, in vivo confocal microscopy (IVCM) has been widely used to observe the morphological changes of corneal subbasal nerve fibers [9], and it has important significance for auxiliary diagnosis of EK. However, few studies have focused on the characteristics and therapy of EK following cataract surgery. Here, we carried out the study to explore more details about EK after cataract surgery in diabetic and nondiabetic patients for better prevention and treatment of the disease.

## 2. Materials and Methods

The study conformed to the requirements of the Declaration of Helsinki and was approved by the Ethics Committee of Qingdao Eye Hospital (2020–37). Written informed consent was obtained from all subjects recruited in this study.

### 2.1. Patients

A total of 89 consecutive patients, including 37 patients with DM (37 eyes) and 52 patients without DM (52 eyes), were diagnosed with EK after cataract phacoemulsification combined with intraocular lens implantation from January 1, 2018, to December 30, 2019. The medical data were retrospectively reviewed. Patients were divided into the diabetic group and the nondiabetic group. All of them had no corneal epitheliopathy or epithelial lesions caused by trauma before cataract surgery.

### 2.2. Corneal Fluorescent Staining

EK was defined as superficial punctate erosion and/or epithelial defects. A wet sodium fluorescein strip was gently placed on the lower eyelid fornix of patients and removed, and the location and progress of corneal epithelial lesions after cataract surgery were observed with fluorescein staining under the cobalt blue light of a slit lamp microscope.

### 2.3. Meibomian Gland Dysfunction (MGD) Staging

The extent and severity of MGD before and after cataract surgery were evaluated by the testing of expressibility and secretion quality of meibomian glands [10]. According to the International Workshop on Meibomian Gland Dysfunction, the examination was performed with moderate digital pressure and a standardized technique. The clinical summary of the MGD staging was as follows [10]: stage 1, minimally altered expressibility and secretion quality, no symptoms, and no corneal staining; stage 2, mildly altered expressibility and secretion quality, minimal symptoms, and none to limited corneal staining; stage 3, moderately altered expressibility and secretion quality, moderate symptoms, and mainly peripheral corneal staining; stage 4, severely altered expressibility and secretion quality, marked symptoms, and marked corneal staining; “Plus” disease, coexisting or accompanying disorders of the ocular surface or eyelids.

### 2.4. Tear Film Function Examination

Preoperatively, the tear film function was evaluated by the Oculus dry eye analyzer (Oculus, Wetzlar, Germany). The meibomian gland atrophy scoring was as follows: score 1, gland atrophy accounting for ≤1/3 of the total; score 2, gland atrophy accounting for 1/3 to 2/3 of the total; score 3, gland atrophy accounting for ≥2/3 of the total. The maximum score was 6. The tear breakup time (BUT) < 10 seconds and the low tear meniscus height (LTMH) < 0.20 mm were both considered abnormal. Measurements were taken three times, and then the average value was recorded.

### 2.5. Cataract Surgery

All surgeries were performed by an experienced cataract surgeon. Each patient received the standard surgical procedure successfully. The incision was located in the superior, horizontal, superotemporal, or superonasal clear cornea. The phacoemulsification energy and time were recorded.

### 2.6. Topical Medications after Cataract Surgery

All patients were given topical medications after cataract surgery, including gatifloxacin eye gel (Diyou^®^, 3 mg/g, 0.01% benzalkonium chloride as preservative; Shenyang Xingqi Pharmaceutical Co., Shenyang, China) three times a day for one week, tobramycin-dexamethasone eye ointment (TobraDex^®^, 0.3% tobramycin and 0.1% dexamethasone, 0.5% chlorobutanol anhydrous as preservative; S. A. Alcon-Couvreur N. V., Puurs, Belgium) once per night for two weeks, bromfenac sodium eye drops (BRONUCK^®^, 1 mg/g, 0.01% benzalkonium bromide as preservative; Senju, Osaka, Japan) twice a day for four weeks, and prednisolone acetate ophthalmic suspension (Pred Forte^®^, 10 mg/g, 0.006% benzalkonium chloride as preservative; Allergan Pharmaceuticals, Westport, Ireland) four times a day for four weeks.

### 2.7. Morphology of Subbasal Nerve Fibers in the Area of Epithelial Defects and Corneal Sensation Testing

Morphology of subbasal nerve fibers in the area of epithelial defects was observed by IVCM performed using the Heidelberg Retina Tomograph II Rostock Corneal Module (Heidelberg Engineering GmbH, Heidelberg, Germany) as previously described [11]. Bilateral corneal sensation was tested by using a Cochet-Bonnet aesthesiometer (Luneau Ophthalmology, Paris, France). The central cornea was stimulated vertically with nylon filament, starting from 60 millimeters in length and gradually decreasing until corneal sensation appeared, where corneal sensation was defined as the corneal perception of the longest silk line. The test was repeated twice, and the average length was obtained.

### 2.8. Therapeutic Approaches to EK

The use of NSAIDs was discontinued when the patients manifested diffuse superficial erosion or epithelial defects. Deproteinized calf blood extract eye gel (Sugaojie^®^, 200 mg/g; Shenyang Xingqi Pharmaceutical Co.) was administered three times a day as a proepithelial repair drug at the early stage of EK. Gatifloxacin eye gel (Shenyang Xingqi Pharmaceutical Co.) was used three times daily for one week. Fluorometholone eye drops (Flumetholon^®^, 1 mg/g; Santen, Osaka, Japan) were administered twice or three times per day as an anti-inflammatory drug.

If the drug treatment was ineffective and the epithelial lesions progressed, amniotic membrane transplantation (AMT) was performed after pathogenic infection was excluded. Human amniotic membrane was placed with the basement side up onto the entire cornea and secured using a continuous 10–0 nylon suture within 1 mm of the limbus and 8 to 10 interrupted sutures at the superficial sclera. The amniotic membrane would be removed if it dissolved or fell off early after surgery. If there was no infection, the membrane would be removed at 2 weeks. The healing time of the epithelium was defined as the number of days from the onset of medical treatment to the negative corneal epithelial fluorescence in staining.

### 2.9. Statistical Analysis

The data were presented as percentage and mean ± SD values. The differences in preoperative tear film function, pre- and postoperative MGD staging, and predisposing factors of EK were analyzed between the two groups using the Chi-square test or the Mann–Whitney *U* test. Then the differences in clinical characteristics and therapeutic effects were further assessed using the Chi-square test and Fisher's exact test. All statistical analyses were performed using SPSS (version 24.0; SPSS, Inc., Chicago, IL, USA). Statistical significance was defined as *P* < 0.05.

## 3. Results

### 3.1. Predisposing Factors Related to EK

The mean age of 89 patients was 69.85 ± 9.61 years (range: 47–83 years).  Table 1 shows the comparison of the preoperative tear film function between the diabetic and nondiabetic groups. The predisposing factors related to EK are presented in Table 2. Topical NSAIDs were used for over 4 weeks after cataract surgery in 83.78% (31/37) of diabetic patients and 36.54% (19/52) of nondiabetic patients due to inflammatory reaction of the anterior chamber. The average time of the use of topical NSAIDs in the diabetic group was 44.69 ± 10.51 days, longer than that in the nondiabetic group (33.35 ± 5.16 days, *P* < 0.001).

The diagnosis and staging of MGD are shown in Table 3. Preoperatively, 83.78% (31/37) of the diabetic patients were diagnosed with MGD, which had significant difference from the nondiabetic patients (*P* < 0.001). Patients at the “Plus” disease stage accounted for 48.65% (18/37) in the diabetic group postoperatively, and the proportion was higher in the nondiabetic group (*P* < 0.001).

### 3.2. Clinical Characteristics of EK

**Figure 1:**
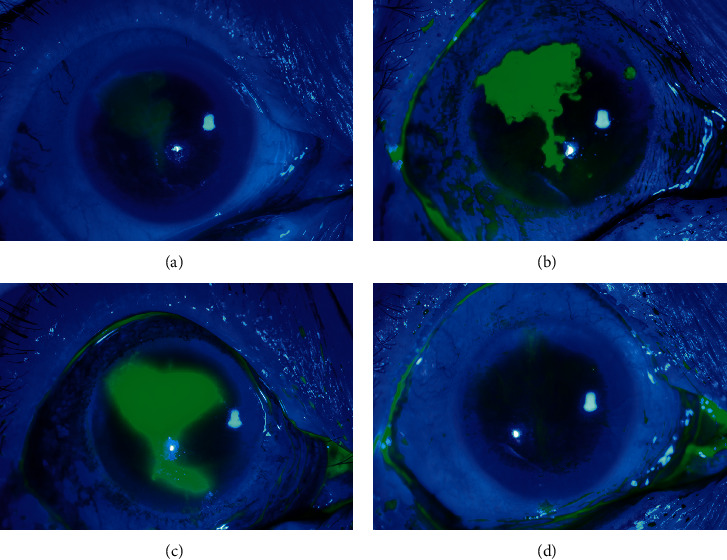
Fluorescence staining photographs of an eye with epithelial keratitis. Progression of an epithelial defect near the temporal clear corneal incision ((a) upon the initial visit; (b) after 4 days; (c) after 10 days). Healing of the corneal epithelial defect after topical antiviral treatment ((d) one month after the initial visit).

Corneal epithelial punctate erosions in 60.67% (54/89) of all patients gradually developed into diffuse epithelial damage and even corneal epithelial defects (34.83%, 31/89).  Table 4 demonstrates the characteristics of EK in the diabetic and nondiabetic groups. Although EK occurred in diabetic patients at 17.62 ± 7.61 days after cataract surgery, which was earlier than in nondiabetic patients (21.63 ± 6.43 days), there was no significant difference between them (*P*=0.336). The proportions of patients suffering epithelial damage progression within three to four days and epithelial defects in the diabetic group were both significantly higher than the nondiabetic group (*P*=0.025, <0.001). Moreover, the healing time of the corneal epithelium in diabetic patients was longer than that in nondiabetic patients (40.62 ± 20.0 days vs. 21.74 ± 6.94 days, *P*=0.002). In the diabetic group, two patients (5.41%) were diagnosed with epithelial HSK on the basis of the clinical signs and response to the antiviral treatment, and the corneal epithelial damage gradually healed following topical antiviral therapy (Figure 1). However, one patient (2.7%) finally developed corneal ulceration.

**Figure 2:**
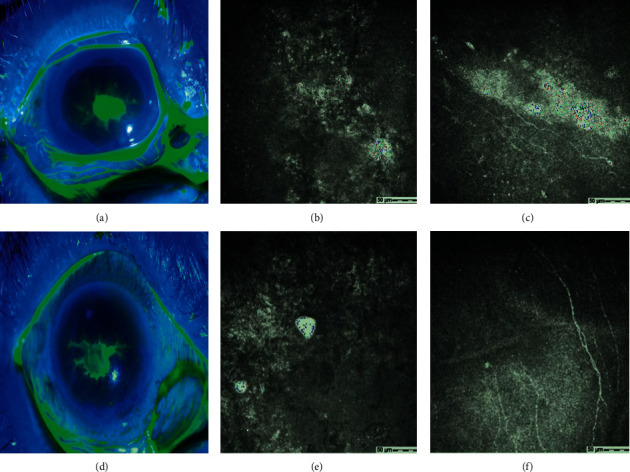
Comparison of patients suffering epithelial keratitis combined with diabetes or not. Fluorescence staining of the corneal epithelial damage in a diabetic patient (a) and a nondiabetic patient (d). Confocal microscopic images from patient (a) showing the stromal edema and absence of subbasal nerve fibers in the area of corneal epithelial lesions (b) before the treatment for keratitis and obviously slender subbasal nerve fibers after the corneal epithelial healing (c). Confocal microscopic images from patient (d) showing the absence of subbasal nerve fibers in the area of corneal epithelial lesions (e) before the treatment for keratitis and sparse subbasal nerve fibers after the healing of the corneal epithelium (f). All confocal microscopic images are in the scale of 400 × 400 *μ*m.

IVCM was performed to observe the morphology of subbasal nerve fibers in 31 patients with epithelial defects. There was corneal stromal edema but no subbasal nerve fibers in the area of corneal epithelial damage (Figures 2(b) and 2(e)) before the treatment for EK, and there were sparse subbasal nerve fibers when the corneal epithelium healed (Figures 2(c) and 2(f)) in both groups. In these patients, central corneal sensitivity was also examined. It was found to be significantly depressed in the contralateral eyes of diabetic patients as compared to the contralateral eyes of nondiabetic patients (5.2 ± 1.03 cm vs. 5.83 ± 0.41 cm, *P*=0.004). However, there was no significant difference in central corneal sensation of the affected eyes between the two groups when the corneal epithelium was damaged (3.8 ± 1.69 cm vs. 4.33 ± 1.47 cm, *P*=0.277).

### 3.3. Therapeutic Effects

Corneal epithelial healing after the treatment of only medications was achieved in 67.57% (25/37) of diabetic patients and 98.08% (51/52) of nondiabetic patients. The proportion of patients requiring AMT was 32.43% (12/37) in the diabetic group, which is higher than the nondiabetic group (1.92%, 1/52; *P* < 0.001). Two (16.67%, 2/12) diabetic patients with persistent corneal epithelial defects underwent AMT more than twice. Although the corneal epithelium healed in all patients at the final follow-up, there were corneal nebulae in 29.73% (11/37) of diabetic patients, and the incidence was higher than that in nondiabetic patients (11.54%, 6/52; *P*=0.031).

## 4. Discussion

Ocular surface diseases may lead to a decreased adhesion of the epithelial basement membrane and thus accelerate the occurrence of EK. MGD, as a major ocular surface disease, gradually increases with age [12]. However, age is not the only reason for MGD. Systemic diseases such as DM could also be associated with it. EK after cataract surgery is not uncommon in clinical practice. In an animal study, EK and other ocular surface diseases were found to result from the abnormal tear function after cataract surgery [13]. As Sangwan et al. [14] reported, surgeries for cataract combined with ocular surface diseases needed careful preoperative, intraoperative, and postoperative planning to prevent postoperative epitheliopathy. In this study, 60.67% of all patients with EK after cataract surgery were more than 70 years old, with an average age of 69.85 ± 9.61 years. Preoperatively, the BUT was <10 s in 91.89% of diabetic patients, and 83.78% of those patients were diagnosed with combined MGD. The tear film function in the diabetic group was poorer than the nondiabetic group. By far, there has been no consensus on topical medications for patients after cataract surgery, although a 1-month treatment regimen is preferred [15]. Due to the inflammatory reaction, it is necessary to use topical NSAIDs for more than one month after surgery for cataract, but prolonged use of eye drops containing preservatives such as benzalkonium chloride may aggravate ocular surface diseases [16] and lead to keratoconjunctivitis medicamentosa due to drug-induced toxicity on the corneal epithelium in a manner depending on time and concentration [17]. Topical administration of eye drops containing different concentrations of preservatives may cause corneal epithelial damage after cataract surgery. However, EK can be caused by varied etiologies at different times. Toxic keratitis often develops within 7 days after cataract surgery [9], and EK associated with cataract surgery occurs within 2 weeks in the absence of dry eye or MGD [18]. In this study, the occurrence of EK was at more than two weeks after surgery in 77.53% of all patients. Therefore, surgical stimulation was not the only cause of corneal epithelial injury after cataract surgery. In our series, 56.18% of all patients used topical NSAIDs for more than one month, yet the usage time of NASIDs in the diabetic group was much longer. The results suggested that MGD and prolonged use of topical NSAIDs were important risk factors for EK after cataract surgery, especially in patients with DM. Clinicians should take measures to protect the ocular surface when eye drops are used for more than 2 weeks.

DM was discovered to be a risk factor for persistent corneal epithelial defects after pars plana vitrectomy [19]. Nishida et al. [1] reported no difference in the incidence of epithelial defects after different cataract surgeries, but the matter of DM was not involved. In the current study, there was no significant difference in age and ultrasound energy and time between diabetic and nondiabetic patients during cataract surgery, whereas the preoperative ocular surface dysfunction was worse in the diabetic group. The incidence of MGD with severe corneal epithelial staining was significantly higher, a larger proportion of the patients progressed to epithelial defects, and epithelial erosions developed faster in diabetic patients. The healing of corneal epithelial lesions was delayed in patients with DM and severe MGD, and corneal ulceration occurred finally in one patient. Although there was no significant difference in the occurrence time of EK between the two groups, the mean time was earlier in the diabetic group. Moreover, the average healing time of the corneal epithelium was much longer in the diabetic group, which was consistent with a report showing that the corneal epithelial healing required a longer time in diabetic patients across all etiologic categories [7]. AMT could promote the corneal epithelial healing in patients with persistent corneal epithelial defects and prevent infection when drug treatment was useless. In this study, 32.43% of diabetic patients received AMT, and 16.67% underwent this intervention more than twice, yet only 1.92% of nondiabetic patients required AMT. Although epithelial lesions healed in all patients, corneal nebulae appeared in 29.73% of diabetic patients. These results indicated that EK after cataract surgery in patients with DM was more likely to develop into severe corneal epitheliopathy and even secondary infection in a short period of time. AMT was effective in preventing severe complications in diabetic patients with persistent epithelial defects.

A decrease of corneal subbasal nerve fibers is typical characteristics of diabetic keratopathy [18, 20] and contributes to the development of diabetic epitheliopathy [21, 22]. In an animal study, we confirmed that corneal epithelial wound delayed healing in diabetic mice due to the reduced corneal nerve fiber density and subbasal nerve plexus [23]. Nevertheless, the mechanism of diabetic corneal epitheliopathy remains a contentious topic. It was reported that the cutting of the corneal stromal nerve through the incision of cataract surgery, decrease of the subbasal nerve density, and lower initial stromal bed nerve density in diabetic patients may predispose them to develop into diabetic keratopathy [7]. Corneas in patients with DM that appear to be free of disease have actually undergone biochemical and ultrastructural changes [24]. Another study disclosed that the number of subbasal nerve fibers decreased one month after cataract surgery in patients without epithelial defects and returned to normal values 8 months postoperatively [25]. A similar research even found that patients undergoing cataract surgery exhibited bilateral alterations of the corneal subbasal nerve plexus [26], which led to the decrease of corneal sensation [27]. However, both of the two studies excluded patients with diabetes. An animal study proved that the corneal epithelial injury healing was faster than the subbasal nerve regeneration [28]. Nevertheless, morphology of regenerated subbasal nerve fibers and changes of corneal sensation in patients with corneal epithelial injury after cataract surgery are still unknown. In our study, confocal microscopic images showed no subbasal nerve fibers in the area of epithelial defects in the two groups. We also preliminarily observed the morphological distribution of subbasal nerve fibers by IVCM when the corneal epithelium healed, finding the regenerative corneal subbasal nerve fibers in the short term of epithelial healing were obviously sparse. The decline in corneal subbasal nerve fibers can lead to corneal hypoesthesia. In diabetic patients with epithelial defects, central corneal sensation of the contralateral eye was significantly depressed and that of the affected eye was more obviously decreased, although with no significant difference from nondiabetic patients with epithelial defects. These results suggested that the severe corneal epithelial injury in diabetic patients after cataract surgery may be related to the changes in the morphology and density of the subbasal never fibers. HSK, with an incidence of 1.8% after cataract surgery [29], is easily misdiagnosed due to atypical clinical manifestations. Yang et al. [29] observed that HSK after cataract surgery initially appeared as dot damage to the corneal epithelium and gradually developed into dendritic damage [29]. Zou et al. [9] reported that corneal epithelial defects and dendritic lesions presented initially in patients with HSK [9]. That is to say, HSK may present various clinical characters at early stage, which relate to corneal nerve injury caused by corneal nerve disruption or postoperative treatment with topical corticosteroids [13]. In our series, two patients in the diabetic group were diagnosed with epithelial HSK. In the report by Zou et al. [9], corneal epithelial damage in one patient with epithelial HSK had epithelial defects near the main incision of cataract surgery and developed into a geomorphic defect and dendritic lesions without antiviral treatment. Thus, the risk of HSK in patients with EK after cataract surgery should not be neglected.

Due to the retrospective nature, there is a limitation in this study. No healthy patients after cataract surgery were included as controls. A prospective investigation with a wider range of patients will further validate the conclusions.

## 5. Conclusions

Diabetes, abnormal tear film function, MGD, and prolonged use of topical NSAIDs are main risk factors of EK after cataract surgery. EK in diabetic patients can progress rapidly to severe corneal epitheliopathy and result in severe epithelial damage and slow repair of the corneal epithelium. AMT is effective for the treatment of persistent epithelial defects associated with such EK. The findings in this study would be helpful for better prevention and treatment of EK following surgery for cataract.

## Figures and Tables

**Table 1:** Comparison of the preoperative tear film function between the diabetic and nondiabetic groups.

	Diabetic group (*n* = 37)	Nondiabetic group (*n* = 52)	*P* value
Age, y			0.447†
<60	5 (13.51%)	3 (5.77%)	
60–70	11 (29.73%)	16 (30.77%)	
>70	21 (56.76%)	33 (63.46%)	
BUT, s			0.517†
<10	34 (91.89%)	45 (86.54%)	
≥10	3 (8.11%)	7 (13.46%)	
LTMH, mm			
≤0.2	8 (21.62%)	4 (7.69%)	0.068†
>0.2	29 (78.38%)	48 (92.31%)	
Meibomian gland atrophy score			<0.001^*∗*^
<2	7 (18.92%)	32 (61.54%)	
≥2	30 (81.08%)	20 (38.46%)	

y = year; s = second; mm = millimeter.  ^*∗*^Chi-square test. †Chi-square test with continuity correction.

**Table 2:** Comparison of predisposing factors related to EK between the diabetic and nondiabetic groups.

	Diabetic group (*n* = 37)	Nondiabetic group (*n* = 52)	*P* value
	Mean ± SD	Mean ± SD	
Age, y	73.72 ± 5.71	66.83 ± 9.92	0.102§
Preoperative BUT, s	5.35 ± 1.93	7.05 ± 1.95	<0.001§
Preoperative meibomian gland atrophy score	1.97 ± 0.78	1.42 ± 0.60	<0.001§
Preoperative LTMH, mm	0.22 ± 0.03	0.27 ± 0.06	<0.001§
Phacoemulsification power, %	9.64 ± 10.55	8.51 ± 8.10	0.921§
Phacoemulsification time, s	31.13 ± 14.76	30.4 ± 16.21	0.947§
Duration of postoperative use of topical NSAIDs, d	44.69 ± 10.51	33.35 ± 5.16	<0.001§

s = second; mm = millimeter; y = year; d = day. §Mann–Whitney *U* test.

**Table 3:** Diagnosis and staging of MGD between the diabetic and nondiabetic groups.

	Before surgery		After surgery		*P* value
	Diabetic group (*n* = 37)	Nondiabetic group (*n* = 52)	Diabetic group (*n* = 37)	Nondiabetic group (*n* = 52)	
Absence of MGD	6 (16.22%)	30 (57.69%)	0 (0%)	0 (0%)	
Presence of MGD and staging					*P*1 < 0.001^*∗*^
1	21 (56.76%)	15 (28.85%)	0 (0%)	0 (0%)	
2	10 (27.03%)	7 (13.46%)	2 (5.41%)	23 (44.23%)	
3	0 (0%)	0 (0%)	8 (21.62%)	5 (9.62%)	
4	0 (0%)	0 (0%)	9 (24.32%)	11 (21.15%)	
˝Plus˝ disease	0 (0%)	0 (0%)	18 (48.65%)	13 (25.00%)	*P*2 < 0.001^*∗*^

^*∗*^Chi-square test. *P*_1_: comparison of patients with MGD before cataract surgery between the diabetic and nondiabetic groups. *P*_2_: comparison of patients at the “Plus” disease stage of MGD after surgery between the diabetic and nondiabetic groups.

**Table 4:** Comparison of clinical characteristics of epithelial keratitis between the diabetic and nondiabetic groups.

Clinical characteristics	Diabetic group (*n* = 37)	Nondiabetic group (*n* = 52)	*P* value
Progress of epithelial damage			<0.001^†^
Dot superficial erosion	4 (10.81%)	31 (59.62%)	
Progress to diffuse superficial erosion	15 (40.54%)	8 (15.38%)	
Progress to epithelial defects	18 (48.65%)	13 (25.00%)	

Location of epithelial lesions at early stage			0.600^†^
Nearby the main incision	1 (2.70%)	3 (5.77%)	
Central cornea	24 (64.86%)	24 (46.15%)	
Inferior cornea	12 (32.43%)	25 (48.08%)	

Time of occurrence for EK, w			0.908^†^
<1	2 (5.41%)	3 (5.77%)	
1–2	7 (18.92%)	8 (15.38%)	
>2	28 (75.68%）	41 (78.85%)	
Time to corneal epitheliopathy progression, d			0.025^†^
≤2	3 (8.11%)	6 (11.54%)	
3–4	22 (59.46%)	16 (30.77%)	
≥5	12 (32.43%)	30 (57.69%)	

Complications			
Corneal ulcer	1 (2.70%)	0 (0%)	0.233^Ψ^
Epithelial HSK	2 (5.41%)	0 (0%)	0.090^Ψ^
Corneal nebulae after epithelial healing	11 (29.73%)	6 (11.54%)	0.031 ^*∗*^

d = day; w = week; cm = centimeter. ^†^Chi-square test with continuity correction.  ^*∗*^Chi-square test. ^Ψ^Fisher's exact test.

## Data Availability

The datasets during and/or analyzed during the current study are available from the corresponding author upon reasonable request.
